# A Fresh Glimpse into Cartilage Immune Privilege

**DOI:** 10.1177/19476035221126349

**Published:** 2022-10-15

**Authors:** Carissa Garrity, Boaz Arzi, Brian Haus, Cassandra A. Lee, Natalia Vapniarsky

**Affiliations:** 1 Department of Pathology, Microbiology and Immunology, University of California, Davis, Davis, CA, USA; 2 Department of Surgical and Radiological Sciences, School of Veterinary Medicine, University of California, Davis, Davis, CA, USA; 3 Department of Orthopaedic Surgery, University of California Davis Medical Center, Sacramento, CA, USA

**Keywords:** cartilage immunology, tissue engineering, MHC, allogenic, immunological rejection, T cell

## Abstract

The increasing prevalence of degenerative cartilage disorders in young patients is a growing public concern worldwide. Cartilage’s poor innate regenerative capacity has inspired the exploration and development of cartilage replacement treatments such as tissue-engineered cartilages and osteochondral implants as potential solutions to cartilage loss. The clinical application of tissue-engineered implants is hindered by the lack of long-term follow-up demonstrating efficacy, biocompatibility, and bio-integration. The historically reported immunological privilege of cartilage tissue was based on histomorphological observations pointing out the lack of vascularity and the presence of a tight extracellular matrix. However, clinical studies in humans and animals do not unequivocally support the immune-privilege theory. More in-depth studies on cartilage immunology are needed to make clinical advances such as tissue engineering more applicable. This review analyzes the literature that supports and opposes the concept that cartilage is an immune-privileged tissue and provides insight into mechanisms conferring various degrees of immune privilege to other, more in-depth studied tissues such as testis, eyes, brain, and cancer.

## Introduction

The high prevalence of degenerative articular cartilage disorders among young and adult patients is a public health concern worldwide. Cartilage loss due to trauma or wear leads to osteoarthritis (OA). Clinical symptoms of OA include pain, joint swelling, and restricted range of motion, causing functional disability and reduced quality of life.^1^ In the United States alone, 1 in 25 working-age adults from 18 to 64 years are limited to arthritis.^2^ Out of this population, almost half experience work limitations, equating to $164 billion in lost wages.^2^ It is predicted that by 2040, doctor-diagnosed arthritic conditions will equate to 78.4 million people.^2^ Cartilage loss in pediatric patients is also increasing in frequency due to a significant rise in sports injuries and is of particular concern given the lifelong disability that subsequently occurs.

Cartilage’s innately poor regenerative capacity^3,4^ and progressively increasing demand for its regeneration have resulted in astonishing developments and achievements in the field of tissue engineering (TE) and regenerative therapies. However, despite advancements in cartilage TE and current clinical transplantation practices, the ability to replace deficient or damaged cartilage remains limited. Traditional methods such as fresh articular cartilage with bone (osteochondral) autografts and allografts are currently used as cartilage replacements in affected patients.^5^

Cartilage autografts are derived from the same individual and are harvested from a non-weightbearing area of the joint. However, the quantity of available cartilage is limited, and autograft collection incurs additional morbidity. Allografts are tissues obtained from a donor of the same species. A common example of allogenic cartilage treatments is allogenic osteochondral implantation (OCI), also called osteochondral allograft transplantation (OAT). The benefits of allografts are in the relative availability of donor tissue and the possibility of an off-the-shelf implant that can be introduced in a single surgery without inducing second-site morbidity in the patient.^6,7^ However, allografts present the risk of disease transmission and eliciting immune rejection.^8-10^ For instance, despite the average 12-year success rate of osteochondral allografts, up to 25% of allografts are failing, and 36% of patients require additional or revision surgeries.^11^ The lack of long-term durability and integration could be due to immunological destruction of the graft. Immunological rejection of a tissue graft is typically prevented by immunologic matching of donor and recipient before transplantation. Surprisingly, unlike solid organ allografts, immunologic matching, also known as major histocompatibility complex (MHC) matching, is not routinely performed with osteochondral allografts.^12^ This is due to the historical concept that cartilage is an “immune-privileged” tissue.^13-15^

Immune privilege is best summarized as a tissue’s specific ability to repress an immune response to implanted tissues or antigens,^16^ while immunogenicity is the ability to provoke the immune response.^17^ Mechanisms governing immune privilege in tissues such as brain,^18^ eye,^19^ placenta,^20^ ovary,^21^ and testis^22^ are continuously explored. However, studies addressing the immune status of cartilage are few, and there is a discrepancy between the evidence presented in the literature and how it is translated to clinical practice. For instance, contradicting the immune-privileged claim is the fact that only autologous but not allogenic chondrocytes are used in current cartilage replacement therapies.^23,24^ In addition, reports comparing the outcomes of allogenic OCI in immunologically matched and mismatched human patients^8^ and in dogs^10^ provide evidence that questions the concept of cartilage’s immune privilege. Specifically, in humans, the long-term outcomes of massive osteochondral implants were better when recipients and donors were immunologically matched.^8^ In dogs, the inflammatory response was much more severe in joints that received an immunologically mismatched allograft.^10^ Similarly, a histological study in rabbits comparing the host response to cartilage autografts, allografts, and xenografts (tissue from another species) reported strong innate and adaptive immune responses to xenografts, mild responses to allografts, and no response to autografts, in osteochondral implants.^13^

In contrast, there are numerous studies that provide strong evidence of immunosuppressive and potentially immune-privileged properties of chondrocytes and engineered cartilage neotissue. For instance, several studies have demonstrated that chondrocytes are not only unable to stimulate immune responses, but they also suppress the proliferation of activated immune cells.^14,25,26^ It is possible that the application of engineered tissue replacements is hindered by our lack of understanding of cartilage immunology and immune privilege. Here, we critically review the literature supporting and confronting the notion that cartilage is an immune-privileged tissue and provide an insight into novel methods that can advance the field of cartilage regeneration.

## MHC as a Major Player in Transplantation

The primary role of the MHC molecules is to assist in the maintenance of an organism’s health by presenting foreign, self-, or altered self-peptides to immune cells (lymphocytes and, more specifically, T cells). The function of MHC molecules was first discovered in the context of transplantation in mice, hence, the name major histocompatibility complex.^27^ However, the primary role of MHC molecules is immunological surveillance and marking of altered or infected cells for the adaptive immune system’s elimination and mounting of the humoral and cellular immune response. The terminology for MHC equivalent in humans identifies the same molecules as (human leukocyte antigen) HLA; in swine as SLA; in a dog, DLA, in cat FLA, and so on for any other species. For this review, the MHC acronym will be used regardless of species. The immune response to non-self-MHC molecules and non-self-peptides presented by the MHC complexes are the primary causes of transplant rejection. For the benefit of understanding the immune status of cartilage tissue, it is imperative first to review the classes and the function of MHC in more detail.

**Figure 1.**
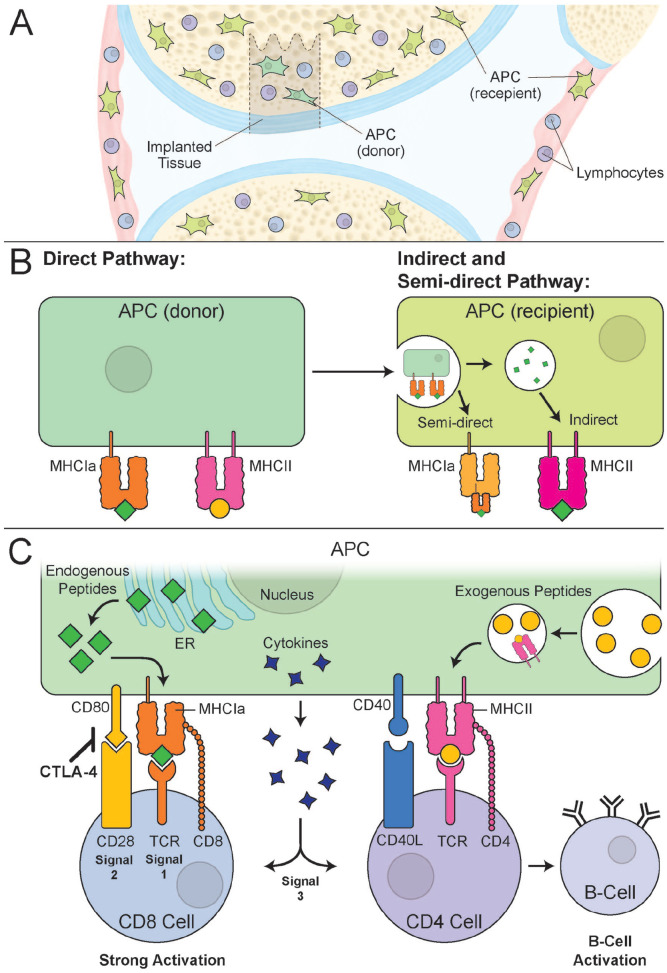
MHC antigen presentation pathways in the context of osteochondral implants. (**A**) In a scenario of osteochondral allograft implantation, APCs can be either donor or recipient derived. The donor APC will reside in the implant’s bone marrow cavity, while recipient APC will be present in the synovium and in the recipient’s bone marrow. (**B**) In the direct pathway of antigen presentation, donor APC will present donor peptides/antigens on MHC class Ia and MHC class II complexes. In the semi-direct pathway, recipient APC can fuse with donor cell or donor cell exosome membrane to present intact donor MHC Ia complexes with associated donor peptides. In the indirect pathway, a recipient APC presents digested donor peptides (green rectangles) on its MHC class II complex. As a result, the recipient APC can present digested donor peptides or whole donor MHC molecules, which will be subsequently recognized by lymphocytes. (**C**) Peptide presented by MHC class Ia will be recognized by a recipient cytotoxic T cell (CD8, T cell) and result in strong activation of the lymphocyte. Peptides presented by MHC class II complex will be recognized by T-helper lymphocytes (CD4, T cell). Besides MHC class Ia or MHC class II recognition, additional signals are necessary to activate CD4 T-helper cells or CD8 cytotoxic T cells. In addition to MHC class Ia with presented endogenous peptide binding to the T-cell receptor (TCR) (Signal 1), there should be an additional binding of co-receptor CD8 with MHC complex and CD28 (T cell) binding with CD80/B7 (APC) (Signal 2). Stimulation with appropriate cytokines secreted by the APC will further aid in the activation of the CD8 T cell (Signal 3). To keep activation in balance, the cytotoxic T-lymphocyte-associated protein 4 (CTLA-4) expression by CD8 T cell will inhibit activation of this cell. A similar mode of 3-signal activation will be needed upon engagement of TCR on the CD4 T-helper cells with exogenous peptides presented by the MHC class II complexes. Upon sufficient activation of CD4 T cells, B cells are activated by recognizing peptides bound to its B-cell receptor and presented by MHC II from CD4 T cells. Once B cells are activated, they will generate antibodies against the peptide recognized by B-cell receptors. MHC = major histocompatibility complex; APC = antigen-presenting cell.

There are 2 classes of MHC molecules. MHC class I molecules fit into 2 categories: classical (MHC Ia) and non-classical (MHC Ib).^28^ The MHC I molecules are expressed on all nucleated mammalian cells. The class II MHC is expressed by professional antigen-presenting cells (APCs), such as B cell, dendritic cells, and macrophages. As MHC I and MHC II play a critical role in the recognition and activation of immune cells by presenting self-, altered-self-, or non-self-peptides (alloantigens), these molecules are the main drivers of transplant rejection. Analogous to personal identification documents, they are located on the surface of the cells and present intracellular or extracellular components. Specifically, MHC II molecules present extracellular peptides taken up by phagocytosis, while MHC I molecules present intracellular peptides.^28^ If the MHC presents foreign or inappropriate peptides, activation and clonal expansion of effector T cells will follow, leading to the destruction of the targeted cells/organs. The peptides MHC molecules present are recognized by T lymphocytes via direct, indirect, and semi-direct pathways (Fig. 1A and **B**). For T cells to be properly activated by MHC, 3 signals should be perceived in conjunction: (1) MHC-peptide complex binding the respective T-cell receptor and co-receptor (CD4 or CD8), (2) binding to co-stimulatory receptors (CD28 and CD80/B7 on lymphocytes and APCs, respectively), and (3) cytokine secretion^28^ (Fig. 1C). Notably, the lack of MHC expression will be interpreted by natural killer (NK) cells as missing self, and cells with no MHC expression will be killed.

**Figure 2.**
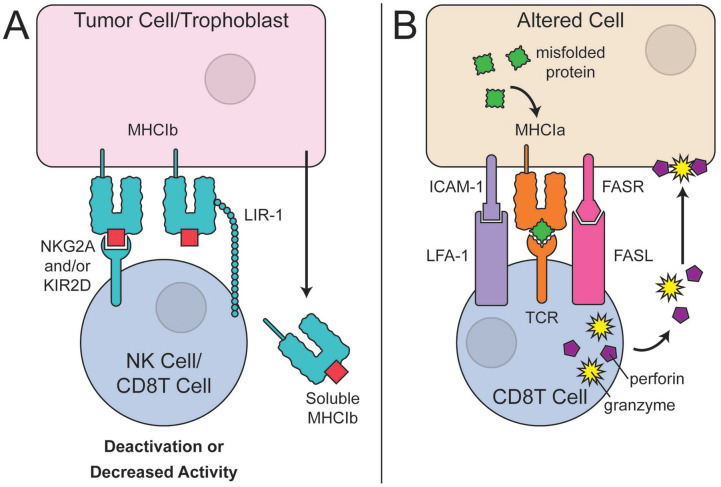
Non-classical MHC and altered-self recognition via classical MHC class I. (**A**) Tumor cells and trophoblasts are known to express the non-classical, MHC class 1b molecules that lead to deactivation and decreased activity of CD8 T cells and NK cells. The binding of minor MHC class 1b complexes is via KIR2D, NKG2A, and LIR-1 receptors. (**B**) In case of neoplastic transformation or other alteration of cells, the signal protein peptide for MHC I synthesis will be altered or mutated which will be readily recognized by CD8 T cells. The binding and subsequent activation of cytotoxic CD8 T cell will result in the release of granzyme and perforin enzymesmediated destruction of the altered cell. The binding of additional co-receptors such as Fas ligand (FasL) with Fas receptor (FasR) and LFA-1 with ICAM-1 on the CD8 T cell and the altered cell, respectively, will further activate the cytotoxic cell. MHC = major histocompatibility complex; NK = natural killer.

The non-classical MHC I molecules (MHC Ib) are far less studied and are thought to play a critical role in regulating immune activation.^29^ Specifically, the non-classical MHC I mediate inhibitory or activating stimuli in NK cells (cells of the innate immune system that are capable of killing other cells without the need for specific antibodies) and cytotoxic T cells.^30^ MHC Ib molecules function as a shield to ensure that the cells are not incorrectly lysed by immune cells.^28^ Non-classical MHC molecules present the leader peptide (or signal peptide, that initiates the process of assembling all the components of MHC I) derived from classical MHC I molecules to lymphocytes.^28^ These non-classical MHC-peptide complexes bind to the inhibitory receptors, NKG2A, KIR2D, and LIR-1 on NK cells or CD8 T cells to prevent the cell lysis^28^ (Fig. 2A). The expression of non-classical MHC molecules is thought to be associated with evasive immune mechanisms seen in tumors,^31^ placental trophoblasts,^32,33^ the eye,^34^ and testis.^35^

When MHC I complexes present a non-self- or altered peptide, cytotoxic T cells recognize the mismatch and destroy the immuno-incompatible cell (Fig. 2B). Although cartilage is considered an immune-privileged tissue, it is unclear if cartilage MHC expression levels and functionality is different compared with other tissues. For example, it would be essential to understand if the immune privilege of cartilage is due to non-classical MHC I components. Based on our knowledge, it is currently unknown if chondrocytes express non-classical MHC molecules. Many aspects of cartilage immunology still need to be explored before the status of cartilage’s immune privilege can be confirmed and fully understood. In the following sections of the review, we will discuss additional aspects of current knowledge of cartilage immunology.

MHC I matching prevents the recognition of donor MHC as an alloantigen (peptide derived from a different individual) and prevents rejection. MHC molecules are highly polymorphic and diverse across individuals. This diversity allows the host’s immune system to recognize a broad range of pathogens (viruses and bacteria). Still, unfortunately, as a side effect, recognition of non-self-MHC results in the mounting of the immune response following transplantation. Determining the degree of similarity of MHC molecules (MHC matching) between donor and recipient before transplantation helps to select individuals with similar MHC phenotypes.^28^ It is done by comparing the blood type and evaluating anti-MHC antibodies in the recipient’s serum.^36^ However, even in MHC identical individuals, rejection can still be facilitated by MHC presentation of other alloantigens. Therefore, recipients may require lifelong immunosuppressive therapy to prevent immunological rejection of the transplant.^28^ Consequently, it is not surprising to find down-regulation of MHC on tumors^37^ or the trophoblast,^38^ tissues that typically avoid immune recognition and subsequent destruction.

## Immune Characteristics of Cartilage

### Evidence Supporting Immune Privilege

**Figure 3.**
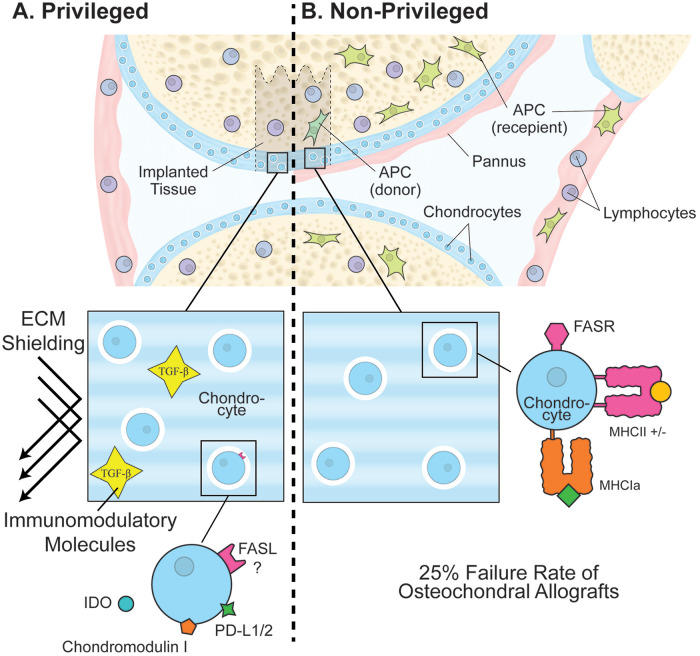
Concepts supporting and refuting cartilage’s immune privilege. (**A**) The left side of the scheme presents factors supporting the privilege. ECM shielding is probably the most common argument. The presence of immunomodulatory molecules such as TGF-β in the ECM is another possibility. Some groups demonstrated expression of Fas ligand (FasL), CD80/B7 inhibitors, such as PD-L1/L2, and secretion of IDO by chondrocytes. (**B**) The right side presents factors disputing the immune privilege of cartilage. Chondrocytes, just like any other somatic cells, express MHC class 1a molecules and may express MHC class II upon inflammatory cytokines stimulation. APC and lymphocytes are present in the synovium of all synovial joints and will be able to detect non-self-antigens. Histological examination of not-matched osteochondral allografts determined the presence of pannus tissue. Pannus formation is usually induced by antigenic stimulation in cases of rheumatoid arthritis or bacterial infections. ECM = extracellular matrix; MHC = major histocompatibility complex; APC = antigen-presenting cell; IDO = indoleamine 2,3-dioxygenase.

Numerous studies have suggested that chondrocytes are immune evasive.^14,25,26,39^ Adkisson *et al.*^14^ showed that isolated chondrocytes inhibit T-cell proliferation *in vitro* through 3 main mechanisms: expression of CD80/B7 inhibitors, chondromodulin I, and secretion of indoleamine 2,3-dioxygenase (IDO). B7 inhibitors, such as PD-L1, directly bind to CD28/PD-1, hence preventing the co-stimulation needed for effector and naïve T-cell activation.^28^ Chondromodulin-I is a glycoprotein that promotes chondrogenesis^40^ and inhibits CD4 T-cell activation by downregulating the production of IL-2.^41^ IDO is an enzyme involved in tryptophan catabolism.^42^ By depleting the extracellular environment from tryptophan, IDO prevents T effector cell proliferation and induces cellular arrest in these cells.^43^ In addition, the metabolites of IDO are toxic to CD8 T cells and Th1 cells, while IDO itself is reported to promote T regulatory cell differentiation.^43^ IDO has been found in immune-privileged tissues such as the testis^44^ and has been shown to play a role in preventing allogenic rejection of fetuses during pregnancy.^45^ However, IDO has also been detected in non-privileged tissues such as the spleen and skin in mice.^46^ Due to IDO expression in both privileged and non-privileged tissue, it would be inaccurate to argue that the presence of IDO alone is sufficient to induce immune privilege (**Fig. 3A**).

An additional argument is that immune-evasive properties of chondrocytes are linked to their mesenchymal lineage.^47-49^ Mesenchymal stromal cells (MSCs) are the progenitor cells to chondrocytes with well-documented immunomodulatory properties.^50^ MSCs inhibit T-cell proliferation^50-52^ through the expression of non-classical MHC molecules and the secretion of soluble mediators such as TGF-β, IL-10, and IDO.^53-56^ Chondrocytes also secrete TGF-β and IDO. Despite the shared lineage between chondrocytes and MSCs, terminal chondrogenic differentiation can alter chondrocytes’ immune characteristics, making them more immunogenic.^57^ For example, rat MSC-derived chondrocytes were shown to acquire the surface expression of CD80/B7 and CD86/CD28, which rendered them more immunogenic than the parent MSCs lacking expression of these molecules.^57^ With that said, human articular chondrocytes have no expression of CD80 or CD86 at either protein or RNA levels.^26^ Species differences could explain the latter. While direct contact between lymphocytes and chondrocytes is possible *in vitro*, in reality, chondrocytes *in situ* are surrounded by a dense network of extracellular matrix (ECM). Thus, the third postulate for chondrocyte immune privilege claims that ECM sequesters antigens and shields against immune detection.^25,58,59^

The composition and density of ECM are essential for the function of the tissue but may also shield chondrocytes from direct contact with lymphocytes. A lymphocyte has an average diameter of 6 to 7 µm.^60^ Wolf *et al.*^61^ have shown that T cells can migrate through multicomponent synthetic lattices by amoeboid shape changes as long as the gap size remains below 1 to 2 µm. During transplant rejection of solid organs, lymphocytes must traverse the basement membrane (a type of ECM).^62^ The pore size of basement membrane is 0.75 to 3.85 µm,^63^ about half the diameter of a lymphocyte. The average pore size of cartilage ECM is 6 nm in diameter,^64^ a thousand times smaller than lymphocyte diameter. The latter suggests that the density of cartilage’s ECM may protect allogenic chondrocytes from lymphocyte detection. It remains to be determined what happens in case of ECM degradation and if lymphocytes can “find” allogenic chondrocytes upon even the slightest ECM breakage.

It is possible that, in addition to the structural barrier, the ECM contributes to the immune privilege of cartilage by harboring non-cellular immune modulators. For example, active and latent forms of TGF-β1 were reported to be present in the ECM.^65^ TGF-β is important in chondrogenesis,^66^ ECM synthesis,^67^ and inducing tolerance through the induction of T regulatory cell differentiation.^28^ High molecular weight hyaluronic acid, a major glycosaminoglycan of cartilage ECM, was reported to induce the expression of regulatory genes such as IL-10 in macrophages.^68^ Therefore, it is reasonable to assume that ECM density, entrapped bioactive proteins, and structural components directly may contribute to immune privilege of cartilage tissue.

Recently, a significant contribution of CD8^+^ T regulatory cells to the immune-evasive mechanisms of immune-privileged tissues^69-72^ was reported. CD8^+^ T regulatory cells have been found in immune-privileged tissues such as the eye^73^ and testis.^74^ The CD8^+^CD122^+^ T regulatory cells are naturally occurring regulatory cells that inhibit the proinflammatory cytokine (IFN-gamma) by activated CD8 and CD4 T.^75^ If CD8^+^ T regulatory cells reside in the joint or in the cartilage tissue, it could explain the lack of immune responses seen following cartilage transplantation.^76^ Further investigation is needed to determine if CD8^+^ T regulatory cells are present in cartilage grafts or within other components of the synovial joint.

### Evidence Confronting Immune Privilege

While the arguments presented in the previous section support cartilages’ immune privilege, they are primarily based on *in vitro* or *ex vivo* experiments. It is unclear if these mechanisms translate to *in vivo* situations where the tissue integrity and joint environment are not controlled experimentally. A 12-year follow-up study of osteochondral transplants in 291 patients reported a 25% failure rate with a 36% rate of revision surgery.^11^ This review did not discuss the reasons for the implant failure.^11^ The authors do not specify if immunological matching was performed between the donors and recipients, but based on the general trend in the field, it is likely that it was not performed. As osteochondral plugs include cartilage, bone, and bone marrow, elements other than cartilage may compromise the successful integration of osteochondral transplants. Indeed, it was established a long ago that fresh and frozen bone allografts are highly immunogenic.^77^ The bone marrow elements were also proven immunogenic.^78^ In support of the latter radiologic and histologic studies investigating the failed osteochondral allografts from the talus reported edema around the implants, progressive cyst formation, irregularities at the subchondral plate, fragmentation, subchondral collapse, and importantly abundance of rejection-associated lymphocytic subsets (**Fig. 3B**).^79^

It is currently recommended practice to pulse lavage the osteochondral allografts to physically remove the residual marrow cells to reduce the potential contribution of immunogenicity by the bone marrow.^80,81^ However, a study by Ambra *et al.*^82^ investigating pulse lavage in removing bone marrow elements showed little efficiency in this procedure. A report by Friedlaender *et al.*^8^ compared the long-term outcomes of large OAT in MHC matched versus mismatched in 29 human patients. This study concluded that there is a benefit in MHC matching, particularly for class II MHC.^8^ Specifically, patients who had failed or fair outcomes (some or significant pain, functional limitation, and need for braces) had more anti-MHC antibodies either before or following the transplantation.^8^ A study by Hunt *et al.*^12^ determined that the presence of anti-MHC class I antibodies in immunologically mismatched recipients of fresh osteochondral allografts was associated with poor graft survival compared with recipients with no such antibodies. Also, allograft size was associated with the outcome.^12^ The larger osteochondral allografts were associated with a higher implant failure rate.^12^ Cumulatively, it can be deducted from the studies above that the high degree of osteochondral implant failure can be due to immune incompatibility of bone and bone marrow components; correspondingly, immunologic matching may reduce the chance of osteochondral implant failure.

However, it remains unclear if cartilage tissue itself is immunologically inert or if it is too contributing to the immunologic process in failing implants. In addition to a tight ECM (discussed earlier), cartilage’s lack of vascularity was proposed to contribute to its privileged immune status. Kandel *et al.*^83^ study evaluating failed osteochondral implants from the knee reported pannus formation, fibrillation, degeneration, and erosion of the chondral portions of the grafts. According to the medical dictionary definition, a pannus is a sheet of inflammatory granulation tissue that spreads from the synovial membrane and invades the joint. The synovial membrane is a highly vascularized structure. It is an immunological guardian of the joint containing phagocytic cells capable of antigen presentation and chemical signaling to recruit additional leukocytes from the circulation.^84^ A study in rats where chondrocytes suspended in hyaluronan were cross-transplanted among rats of 2 separate strains found that lymphocyte infiltration was seen not at the articular surface but the deep margin of the implant. The authors suggested that allogenic chondrocytes may have specific surface antigens that may attract leukocytes and NK cells from the bone marrow.^85^ Pannus formation was not reported in this study. Still, the lack of vascularity in the cartilage tissue did not protect it from the egress of leukocytes via other vascularized elements of the joint such as synovium or bone marrow. In concert, these observations suggest that cartilage’s lack of vascularity is not a sufficient mechanism to protect it from immune cells of the other vascularized compartments of the joint and that cartilage is potentially also immunogenic.

Reports from additional animal studies further subvert cartilage’s immune-privilege status. For example, we found no reports on cartilaginous or osteochondral allotransplantation in veterinary surgical clinical practice, despite abundant reports on successful allografting of cartilage under experimental preclinical settings.^86,87^ A study in dogs found that the inflammatory response was the most severe in joints implanted with immunologically mismatched osteochondral allografts.^10^ Also, pannus formation was typical in cases where fresh mismatched implants were introduced.^10^ The same study also reported that cryopreservation of the osteochondral implants enhanced the deleterious effects on the allografts.^10^ Assuming cartilage is immunogenic, it would be essential to analyze what components may potentially contribute to the immunogenicity—cells or ECM.

Studies on the immune response toward decellularized natural biomaterial matrixes that are essentially comprised of ECM molecules indicate that structural molecules can be immunogenic.^88^ Similarly, renal transplantation studies report antibodies to ECM components such as perlecan, fibronectin, and collagens in patients with chronic renal rejection.^89,90^ More specific to cartilage tissue, it was shown by Klatt *et al.*^91^ that nonfibrillar collagen type II enhanced gene expression of proinflammatory mediators and proteolytic enzymes in chondrocytes. Also, hyaluronic acid, a second major ECM component of cartilage, was shown to have differential signaling based on its molecular weight. Specifically, Rayahin *et al.*^68^ demonstrated that contrary to high molecular weight hyaluronic acid that has immunomodulatory properties, low molecular weight hyaluronic acid induced proinflammatory genes in macrophages. The latter 2 studies suggest that cartilage is shielded from immune system surveillance as long as its matrix is intact. However, the immune system reacts when ECM components and chondrocytes are exposed to immune cell-bearing joint elements such as synovium or bone marrow.

Chondrocyte surface antigens, such as MHC molecules, could further contribute to the aggravation of the immune response. It is well known that chondrocytes constitutively express MHC I and, in some species, also MHC II molecules.^25^ Studies by Hunt and Friedlaender (mentioned above) documented the presence of anti-MHC antibodies in patients with poor osteochondral transplantation outcomes.^8,12^ However, it remains to be determined if these antibodies were developed in response to MHC molecular presentation by chondrocytes or other elements of the osteochondral implant. Furthermore, it remains to be determined if and how antibodies contribute to implant failure or negative outcomes. Indeed, Williams *et al.*^92^ describe a mechanism known as accommodation, where the transplant can remain uninjured despite the presence of anti-MHC antibodies directed against it. Several studies confirm this phenomenon may be true in renal^93^ and heart^94^ transplantation. Dehoux and Gianello^95^ suggest that there may be a “delicate balance” between the production of neutralizing and non-neutralizing antibodies to prevent rejection while inducing accommodation. Identifying factors that would tip the balance in favor of accommodation would be an essential contribution to our understanding of cartilage immunology and the ability to engineer and direct outcomes of allotransplantation.^96-98^

## Lessons from Other Immune-Privileged Tissues

Two tissues commonly studied for their immune-privilege mechanisms are the testis^99^ and the eye.^100^ A recent review defined the immune privilege of the testis as a tissue capable of inducing a systemic tolerance of alloantigens, xenoantigens, and immunogenic autoantigens.^99^ The Sertoli cell barrier and a specific macrophage phenotype in the interstitium contribute to the testis’s immune privilege.^99^ A specific phenotype of immunomodulatory interstitial testicular macrophages was identified in rats.^101^ These macrophages differed from the circulating cells by their ability to constitutively produce and secrete anti-inflammatory cytokines.^101,102^ It remains to be determined if similar macrophages are present in the cartilaginous joint.

The expression of transmembrane molecule Fas ligand (FasL) by ocular^100^ and testicular^99^ macrophages are additional factors that contribute to the immune privilege of these organs. The Fas receptor (FasR)-FasL interaction is a signal transduction pathway that regulates cell death.^103^ The cell bearing the receptor is targeted for death, while the cell bearing the ligand induces death.^103^ If a cell expresses the FasR, the cell is capable of being recognized and killed by immune cells. However, if the cell expresses FasL, it can induce apoptosis of immune cells and escape the destruction. While FasR presence on chondrocytes is unequivocal, FasL expression is controversial and was undetectable in healthy and osteoarthritic human cartilage by the Hashimoto group,^104^ but was reported by Fujihara *et al.*^76^ in human and murine auricular chondrocytes. Interestingly, a significant amount of soluble FasL was found in the synovial fluid of OA and rheumatoid arthritis patients.^105^ It remains to be elucidated if the soluble FasL in the synovium contributes to immune-modulatory properties seen in cartilage tissue. Although, the self-perpetuating nature of OA^106^ does not support this notion.

**Figure 4.**
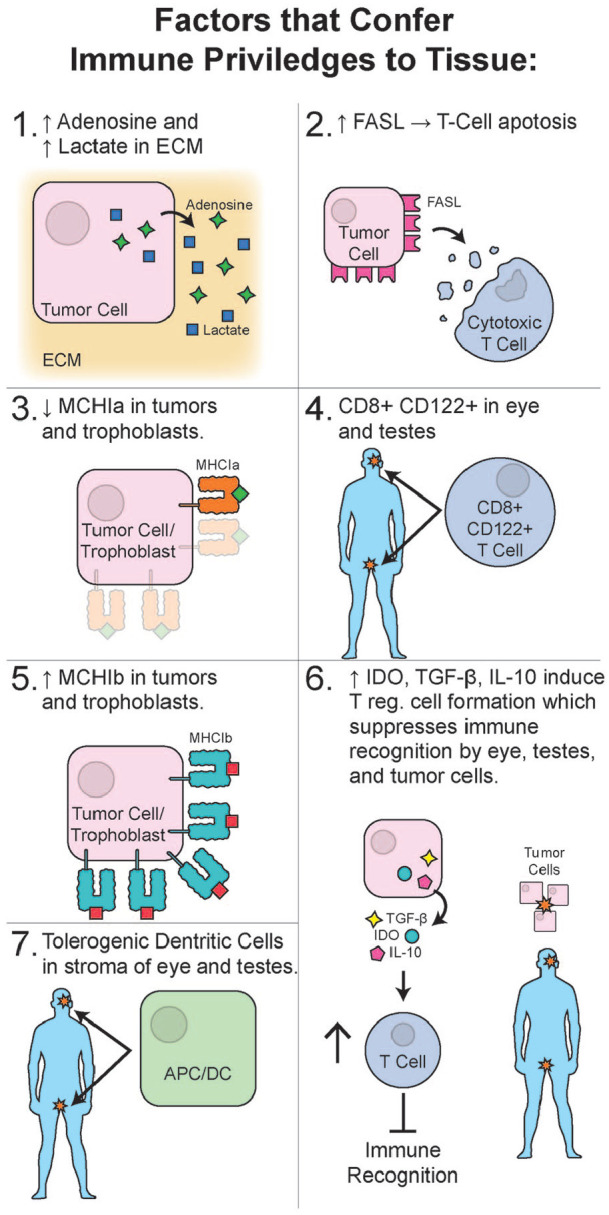
Selected factors known to confer an immune privilege to tissues are shown: (1) Secretion of an increased concentration of adenosine and lactate in the ECM of the tumors; (2) overexpression of FasL by tumor cells was shown to induce apoptosis in cytotoxic T cells; (3) down-regulation of MHC class 1a was reported in regular and transmissible tumors as well as trophoblasts; (4) presence of stromal regulatory T cells CD8+CD122+ was reported in testis and the eye; (5) overexpression of minor, MHC 1b complexes was reported in trophoblasts and tumor cells; (6) secretion of IDO, TGF-β, and IL-10 is a well-documented strategy to induce the T regulatory lymphocytes’ formation to suppress the immune recognition by eye, testis, and neoplastic cells; (7) presence of tolerogenic dendritic cells in the stroma of the eye and testis was reported. ECM = extracellular matrix; MHC = major histocompatibility complex; IDO = indoleamine 2,3-dioxygenase.

Extensive reports exist on cancer immuno-evasion.^107,108^ Selected examples of strategies employed by cancer cells to avoid recognition of altered-self include down-regulation of MHC Ia,^109^ expression of FasL,^110^ and the presence of tolerogenic tumor-associated APC^111^ in the tumor interstitium (Fig. 4). To the best of our knowledge, tolerance-inducing phenotypes of APCs have not been detected in cartilage tissue or the synovium. Further research is needed to characterize leukocyte populations within synovium and their influence on articular cartilage immunology. In addition, there have been no experiments inducing the systemic tolerance to xenoantigens or alloantigens transplanted directly into cartilage or the joint. Therefore, more studies are necessary to determine if the cartilage is indeed an immune-privileged tissue and what factors contribute to this privilege. Perhaps evaluating cartilage immune privilege in the context of the entire joint considering it an organ, would be insightful.

## Methods to Manipulate Immunity

In principle, even with MHC matching for organ transplantation, immunosuppression is still required to avoid rejection.^112,113^ Regrettably, immunosuppression may lead to significant complications such as increased susceptibility to opportunistic infections.^114^ Thus, current research efforts are directed at the investigation of methods of masking recognizable antigens or manipulating genes responsible for immune recognition of non-self-antigens to offer a potential solution for manufacturing immunouniversal organs.^115^

In this context, betta 2 microglobulin (B2M) gene was knocked out in mice resulting in a complete absence of MHC Ia surface molecule expression, making these animals a universal organ donor.^116^ MHC-null pigs^117^ and individual specific cell lines such as hematopoietic blood progenitors and endothelial cells^118^ were developed using a similar strategy. Along these lines, immuno-engineered MHC-null embryonic stem cells^119^ and induced pluripotent stem cells^120^ were explored as promising cell sources for regenerative medicine, considering their unlimited proliferation capacity and the ability to differentiate into any functional tissue.

Although elimination of MHC expression renders cells invisible to lymphocyte recognition, NK cells can detect and destroy cells with “missing self.”^28^ In addition, slight differences in minor histocompatibility molecules or tissue-specific antigens may result in immune rejection.^28^ This problem can be solved by the induction of expression of single-chain dimers of non-classical MHC I complex.^121^ These single-chain fragments of MHC I proteins bind to inhibitory receptors such as CD94/NGK2A on NK cells and T cytotoxic T cells and prevent the cell from lysis. Indeed, this strategy was applied and proved effective in protecting induced pluripotent human stem cells genetically edited to express no MHC Ia but single-chain non-classical MHC Ib from lysis. This study utilized recombinant adeno-associated virus gene editing.^121^

Genetic deletion of MHC I is not the only way to manipulate immunity. Mechanisms that disrupt cytokine gradients and cytokine receptors responsible for effector T cell trafficking can be considered. Expectedly, nature mastered this approach in the maternal-fetal interface where Ccl5 and Cxcl9/10 genes are epigenetically silenced.^122^ As a result of this epigenetic silencing, effector cells cannot reach the fetus, thus preventing its recognition as non-self and subsequent distraction. In the context of cartilage, CCR2 (cytokine receptor) agonist administration to osteoarthritic mice attenuated macrophage accumulation in the synovium, reduced synovitis, and cartilage damage.^123^ This same study showed no benefit of blocking CCL5/CCLR5 axis in attenuating OA. These findings imply that T-cell trafficking signals may be specific to tissue types, and cytokine trafficking manipulation for immunomodulation purposes may need to be tailored to specific tissue types.

Inhibition of cytotoxic T-cell activity, induction of T regulatory cell phenotypes, and shift of macrophage and dendritic cells toward tolerogenic phenotypes are additional tactics that were shown success in immunomodulation. As shown in Figure 1 of this review, CTLA-4 is an inhibitory receptor, also known as CD152, expressed by T regulatory cells or effector T cells after activation that binds to CD80 or CD86 receptors on APC to induce an inhibitory signal. Administration of CTLA-4 immunoglobulins or tetra dimers thereof was shown to be effective in modulating the immune response in patients and animal models of rheumatoid arthritis.^124^ To overcome the challenges of delivery of such potent molecules to the implantation site, nanotechnology and nanoengineering have very clever methods to offer.^125,126^ For instance, to prevent the rejection of xenogeneic pancreatic islets, encapsulating these islets into a nanobot capable of a sustained release of CTLA-4 peptide was shown to prolong pancreatic islet survival in the xenogeneic transplantation model.^127^ CTLA4 biopatterned implant not only survived the transplantation and sustained normoglycemia in the experimental mice but also induced T regulatory differentiation at the implantation site.^127^

Induction of T regulatory phenotypes at the implantation site can be a more precise and accurate way to induce tolerance if APCs are given the correct type of antigen to present to T cells and “educate” these cells to be tolerant toward this particular antigen. The antigen is known in instances like pancreatic islet transplantation or autoimmune destruction. Hence, using dual nanoformulation delivery, impressive work has been done on inducing antigen-specific tolerance and preventing autoimmune type I diabetes in mice.^128^ Specifically, a delivery system comprised of 4 different nanoparticles was designed. Each particle was loaded with either tolerance-inducing factors (Vitamin D3 or TGF-β1) or dendritic cell colony-stimulating factor (GM-CSF), or insulin (antigen). These particles prevented the development of autoimmune destraction of pancreatic islets in experiemntal non-obese diebetic mice model, when administered as a cocktail.^128^ These particles prevented the development of autoimmune destruction of pancreatic islets in experiemntal non-obese diebetic mice model, when administered as a cocktail.^128^ In the context of cartilage, it is unknown if and which peptides may be immunogenic. Thus, more work needs to be done to identify these potentially antigenic molecules before immunoneutral cartilage tissue can be engineered.

## Conclusions

The increasing prevalence of degenerative cartilage diseases is a growing public health concern worldwide. The transplantation of cartilage allografts is regarded as a safe solution due to the historical concept of cartilage being an immune-privileged tissue. However, several studies confront the concept of the immune-privilege status of cartilage by providing evidence of an immune response following allogenic cartilage transplantation. These contradictory findings highlight the need for studies that explore the immunology of cartilage.

The study of immune-tissues is highly instructive in developing universal tissues suitable for transplantation. The immune evasion mechanisms discovered thus far have contributed toward engineering immunologically invisible cell lines. The efficacy of MHC engineering and CRISPR multiplexing in various cell lines suggests that this technology could be translated to cartilage tissue. The application of this technology to cartilage tissue could create universal cartilage allografts that could potentially promote the clinical application of tissue-engineered cartilage and help millions of patients.
